# Schistosomiasis Interventions in Africa: Assessment and Systematic Review

**DOI:** 10.1155/japr/2125107

**Published:** 2025-08-13

**Authors:** Christopher Yaw Dumevi, George Boateng Kyei, Patience B. Tetteh-Quarcoo, James-Paul Kretchy, Irene Ayi, Patrick F. Ayeh-Kumi

**Affiliations:** ^1^Department of Medical Microbiology, College of Health Sciences, University of Ghana Medical School, University of Ghana, Korle-Bu, Accra, Ghana; ^2^Department of Physician Assistantship Studies, School of Medical Sciences, Central University, Accra, Ghana; ^3^Department of Medicine, Washington University School of Medicine, St. Louis, Missouri, USA; ^4^Department of Virology, Noguchi Memorial Institute for Medical Research, College of Health Sciences, University of Ghana, Accra, Ghana; ^5^Medical and Scientific Research Centre, University of Ghana Medical Centre, Accra, Ghana; ^6^Department of Public Health, School of Medical Sciences, Central University, Accra, Ghana; ^7^Department of Parasitology, Noguchi Memorial Institute for Medical Research, College of Health Sciences, University of Ghana, Accra, Ghana

**Keywords:** Africa, nonpharmacological intervention, pharmacological intervention, schistosomiasis

## Abstract

**Background:** Schistosomiasis is a neglected tropical disease with high endemicity across Africa. As a waterborne parasitic disease, the population at highest risk includes school-age children, although adults are also affected. The rationale for this review is to assess the effectiveness of the various schistosomiasis control interventions implemented across Africa.

**Methods:** A targeted systematic search for studies published from January 2000 to August 2023 in African Journals Online, ScienceDirect, PubMed, World Health Organization Database, Cochrane Library and Web of Science databases was conducted. The Preferred Reporting Items for Systematic Reviews and Meta-Analyses (PRISMA) was followed in the screening of the studies conducted from 2000 to 2023.

**Results:** A total of 165 articles (out of an initial number of 9791) that met the inclusion criteria were reviewed in this study under the broad subthemes: pharmacological and nonpharmacological interventions. Praziquantel is the most widely implemented control measure for both preventive and curative purposes across the 35 countries surveyed in this study. Praziquantel either was the sole control strategy (18/35; 51.4%) or was used in conjunction with one or more other interventions (5/35; 14.3%). Studies conducted in 14 countries did not specify the type of schistosomiasis interventions used. Research on schistosomiasis in Africa and its control measures is primarily funded and supported by the WHO and other international research initiatives (49.1%), national governments (17.6%) and private researchers (33.3%). Ineffective coordination at the local, national, regional or continental levels; inconsistent and donor-driven mass drug administration and lack of an effective approach that integrates pharmacological and nonpharmacological control strategies are major bottlenecks hindering the elimination of schistosomiasis across Africa.

**Conclusion:** There is a paucity of data on a systematic approach by the national governments of Africa that effectively integrates pharmacological and nonpharmacological control strategies to meet the 2030 elimination roadmap targets.

## 1. Background

Schistosomiasis, also known as bilharziasis, is caused by the blood-fluke (trematode) belonging to the genus *Schistosoma* [1]. Several species of the genus cause intestinal or urogenital schistosomiasis in humans and animals. *Schistosoma haematobium* causes human urogenital schistosomiasis, whilst *Schistosoma mansoni*, *Schistosoma japonicum*, *Schistosoma intercalatum*, *Schistosoma mekongi* and *Schistosoma guineensis* cause intestinal schistosomiasis [2].

### 1.1. Epidemiology of Schistosomiasis

The acute and chronic nature of the disease affects the general well-being and health of infected individuals. Globally, the burden of *Schistosoma* spp. infection is estimated at 230 million people, with at least 218 million estimated to require treatment and an estimated cost of 8 million disability-adjusted life years (DALYs), over 700 million at risk of infection and also responsible for 534,000 deaths [3]. DALY is a measure of overall disease burden, expressed as the number of years lost to ill health, disability or early death. The World Health Organization (WHO) revealed a global reduction in the number of DALYs lost due to schistosomiasis from 4 to 1.9 million between the years 2000 and 2019. However, schistosomiasis remains endemic in many African countries, and new infections and reinfections amongst the target populations continue to be recorded. An estimated 85% of global schistosomiasis cases are reported in Africa [4].

### 1.2. Interventions and Success

Findings from the study indicated a lack of sustained control interventions; gaps in knowledge, attitudes and practices were factors accounting for increased transmission of the disease in the area. The effective control and possible elimination of schistosomiasis and other neglected tropical diseases (NTDs) could be achieved through multisectoral and integrated approaches [5–7]. Some of the *Schistosoma* species that infect mammals include *Schistosoma bovis*, *Schistosoma curassoni*, *Schistosoma indicum*, *Schistosoma mattheei*, *S. intercalatum* and *Schistosoma hippopotami*. *S. bovis* is one of the main species of veterinary and zoonotic importance. In West Africa, *S. curassoni* and *S. bovis* are responsible for causing schistosomiasis in small animals (sheep and goats) and cattle, respectively [2, 8]. Animal schistosomiasis has also been reported in cattle and pigs in southern Ghana with varied prevalence rates (0.2%–21.7%) in cattle and 0.4% in pigs. A study conducted in Cote d'Ivoire detected a prevalence of 12.5% *S. haematobium–S. bovis* hybrid amongst schoolchildren [9]. Whilst *S*. *haematobium*–*bovis* and *S*. *haematobium*–*mattheei* hybrids have been identified from ova excreted by infected children in Malawi [10], *S. guineensis–S. haematobium* hybrids were identified in Cameroon [11].

These occurrences challenge the current mode of transmission and the infection cycle of the disease since *S. haematobium* has a discrete nonoverlapping infection cycle with animal schistosomes or urogenital and intestinal schistosomiasis coinfection [12]. The hybridization and transmission of human and animal schistosome species is an emerging public concern [13, 14]. The One Health approach, which directly targets the human–animal–environment interface, would involve the implementation of a pharmacological strategy; provision of adequate water, sanitation and hygiene (WASH) facilities; behaviour change communication and health education in endemic areas. Control of freshwater snails by mollusciciding and other approved methods, safe disposal of animal faecal matter, rearing of animals in snail-free areas, treating infected animals and administering preventive chemotherapy (PC) using praziquantel (PZQ) as well as environmental re-engineering are but a few interventions that could disrupt transmission [15].

Over the years, repeated doses of PZQ in endemic and hyperendemic areas have been considered the most effective method of controlling schistosomiasis globally [16]. Schistosomiasis is transmitted in about 78 countries worldwide and is classified as one of the NTDs endemic in resource-poor settings in Africa, South America and Asia where WASH conditions are inadequate [17, 18]. The WHO on November 12, 2020, submitted a new roadmap from 2021 to 2030 aimed at preventing, controlling, eliminating or eradicating NTDs, including schistosomiasis. This roadmap was subsequently launched on January 28, 2021, setting the stage for global targets, programmes and action plans to refocus and align the work of countries, partners and key stakeholders to meet the Sustainable Development Goal (SDG) 3, to which NTD elimination is directly linked [19]. The roadmap for the elimination of schistosomiasis is twofold: the elimination of schistosomiasis as a public health problem by achieving < 1% proportion of heavy-intensity infections and the interruption of transmission (elimination) with the view of reducing to zero the incidence of infection in a defined geographical location with minimal risk of reintroduction, using an integrated approach. The World Health Assembly (WHA) resolution 65.21, adopted in May 2012, urged member states to adopt an integrated approach to eliminate schistosomiasis [18]. In this context, control measures should prioritize effective PC, snail control and the provision of safe and adequate WASH facilities in endemic areas. Additionally, the WHO has introduced operational manuals for the field and laboratory use of molluscicides for schistosomiasis control, intended for programme managers [20, 21]. The WHO's schistosomiasis control strategies in most African countries missed target dates may be due to inadequate intervention coverage from weak health systems, funding gaps, persistent transmission hotspots, competing health priorities and overreliance on mass drug administration (MDA) without complementary measures.

### 1.3. Infection Pathway

Schistosomiasis has a complex transmission pathway involving the passage of eggs in stool or urine from an infected individual into freshwater bodies containing suitable intermediate snail hosts such as the *Bulinus* spp. *or Biomphalaria* spp. The eggs hatch and release miracidia, which invade the freshwater snail hosts. *S. haematobium* penetrates *Bulinus* spp., whereas *S. mansoni* penetrates *Biomphalaria* spp. or, occasionally, *Bulinus* spp. [22, 23].

Upon successful infection of the freshwater snail by the miracidia, they develop into sporocysts and are subsequently released as cercariae, the infective form of the parasite to susceptible hosts. Hundreds of cercariae are shed daily depending on the freshwater snail species involved; for *S. haematobium*, each infected snail host sheds about 200 cercariae, whilst for *S. mansoni*, between 250 and 600 are similarly released [22].

### 1.4. Risk Groups

Humans get infected when they come into contact with infested freshwater during routine agricultural, recreational, occupational or domestic chores. Individuals with increased risk of schistosomiasis include school-age children, women, freshwater fishermen, farmers and irrigation workers. Improper disposal of human faecal waste, open defecation and urinating into freshwater bodies contribute to the increased transmission of human schistosomiasis [17]. Girls of school age are equally at risk of schistosomiasis. Female genital schistosomiasis (FGS) is a common but neglected gynaecological presentation of *S. haematobium* infection in endemic settings [24]. This is occasioned by the deposition of eggs in the female genital tract that causes various symptoms including bleeding disorders, tumours, ulcers and infertility [25]. In endemic areas, blood in the urine of girls at puberty who may not be menstruating is often misdiagnosed as menstrual blood, spotting or a secondary sexual characteristic rather than a potential health issue requiring medical attention [26]. Without early and adequate medical attention, adverse reproductive health outcomes may occur such as preterm labour, ectopic pregnancy and dyspareunia [27].

Schistosomes have an average lifespan of between 3 and 10 years and up to 40 years in permanent copulation within the human host. This leads to chronic disease condition in individuals, some of whom may be asymptomatic reservoir hosts and potential sources of infection transmission [28, 29]. The ecological distribution of the intermediate snail hosts determines the burden of local transmission in an area. Ponds, slow-flowing rivers, lakes, irrigation systems and dams are common water bodies inhabited by the intermediate snail hosts [30]. Depending on the *Schistosoma* spp. involved, the cercariae stage of the parasite penetrates the mucosa or skin, losing its tail and establishing itself in the blood vessels of the bladder or intestines of humans after maturation into adult worms and pairing in the liver.

### 1.5. Clinical Presentation

Clinical presentation of schistosomiasis includes both acute and chronic symptoms. Acute schistosomiasis is characterized by cercarial dermatitis and Katayama fever. Cercarial dermatitis is characterized by a short-term antibody-mediated hypersensitivity reaction as a result of the penetrating and maturing cercariae. This causes an urticarial rash which occurs hours after exposure to cercariae-contaminated freshwater accompanied by fever and cough depending on the infecting *Schistosoma* spp. [1, 31]. Katayama fever, a more systemic hypersensitive reaction, occurs between 14 and 84 days after the cercariae penetrate the skin and the larvae migrate within the body. Katayama fever is generally misdiagnosed in nonendemic areas due to a nonspecific and temporal delay in clinical presentation which may include cough, headache, nocturnal fever, myalgia and abdominal tenderness [31, 32].

Chronic schistosomiasis may occur due to the trapping of the eggs of *S. mansoni* in the intestine or liver or by eggs of *S. haematobium* in the bladder and urogenital system of humans which then triggers immunopathological responses leading to clinical manifestations of hepatosplenomegaly, liver fibrosis or urogenital diseases [1]. Severe anaemia, ureteric stricture, hydronephrosis, stunted growth and low cognitive development and carcinoma of the bladder have all been reported in human schistosomiasis [33].

### 1.6. Speciesism and Hybrid

Although eight sister *Schistosoma* species are found within the *S. haematobium* group, *S. guineensis* and *S. intercalatum* are responsible for intestinal schistosomiasis; *S. bovis*, *S. curassoni* and *S. mattheei* infect livestock, whilst the other species infect wildlife. Infrequent infection and related disease are associated with *S. mattheei* [34]. Conversely, *S. mansoni* has only one sister species, *Schistosoma rodhaini*, which characteristically is found in small rodents with the capacity to hybridize with *S. mansoni* under favourable conditions [34, 35].

Schistosome hybrids may have consequences for treatment outcomes and sustained transmission of schistosomiasis. In Africa, there is a dearth of literature on coinfection of intestinal and urogenital schistosomiasis despite evidence to the contrary [36]. An intriguing set of plausible worm pairings occurs within a coinfected definitive host. This comprises a heterospecific (*S*. *mansoni* [♂] and *S*. *haematobium* [♀]) and homospecific (*S*. *haematobium* [♂] and *S*. *haematobium* [♀]) schistosome couplings. Mating preference, worm competition and specific anatomical site—which could be the vasculature of the hepatoportal, intestinal or urogenital systems—are key factors that determine cross-specific couplings [35, 37]. The possibility of some heterospecific worm pairings is especially significant for currently known genetic introgression involving *S. haematobium* and *S. intercalatum* or, more importantly, as yet unknown interactions (amongst several *S. haematobium* hybrids themselves with or without *S. haematobium* or *S. mansoni* couplings) as more hybrid variants become apparent [10]. Interspecies hybrids of schistosomes are currently being reported in Central Africa and Malawi where *S*. *haematobium*–*bovis* and *S*. *haematobium*–*mattheei* combinations have been identified from ova recovered from infected children [10, 38, 39]. Genomic analysis of a few schistosome eggs retrieved from the schoolchildren in Malawi showed allopatric, geographically distinct distribution of *S*. *haematobium*–*mattheei* and *S*. *haematobium*–*bovis* hybrids even though individual hybrids were sympatric and geographically synonymous with *S*. *mansoni* and *S*. *haematobium* [38]. Although genomic examination and analysis of *S*. *haematobium*–*bovis* hybrid is yet to be conducted, analysis of *S*. *haematobium*–*bovis* hybrids from West Africa showed large 100 kb identical chromosomal regions. This is suggestive of a single or very limited number of hybridization events granted probable multiple rounds of meiosis [40].

With multispecies coinfections occurring in Malawi and Central Africa as a result of heterospecific and homospecific worm pairing couplings, it remains unclear the level of genetic interactions ongoing in schistosomes across Africa and the clinical implications of this phenomenon.

**Figure 1:**
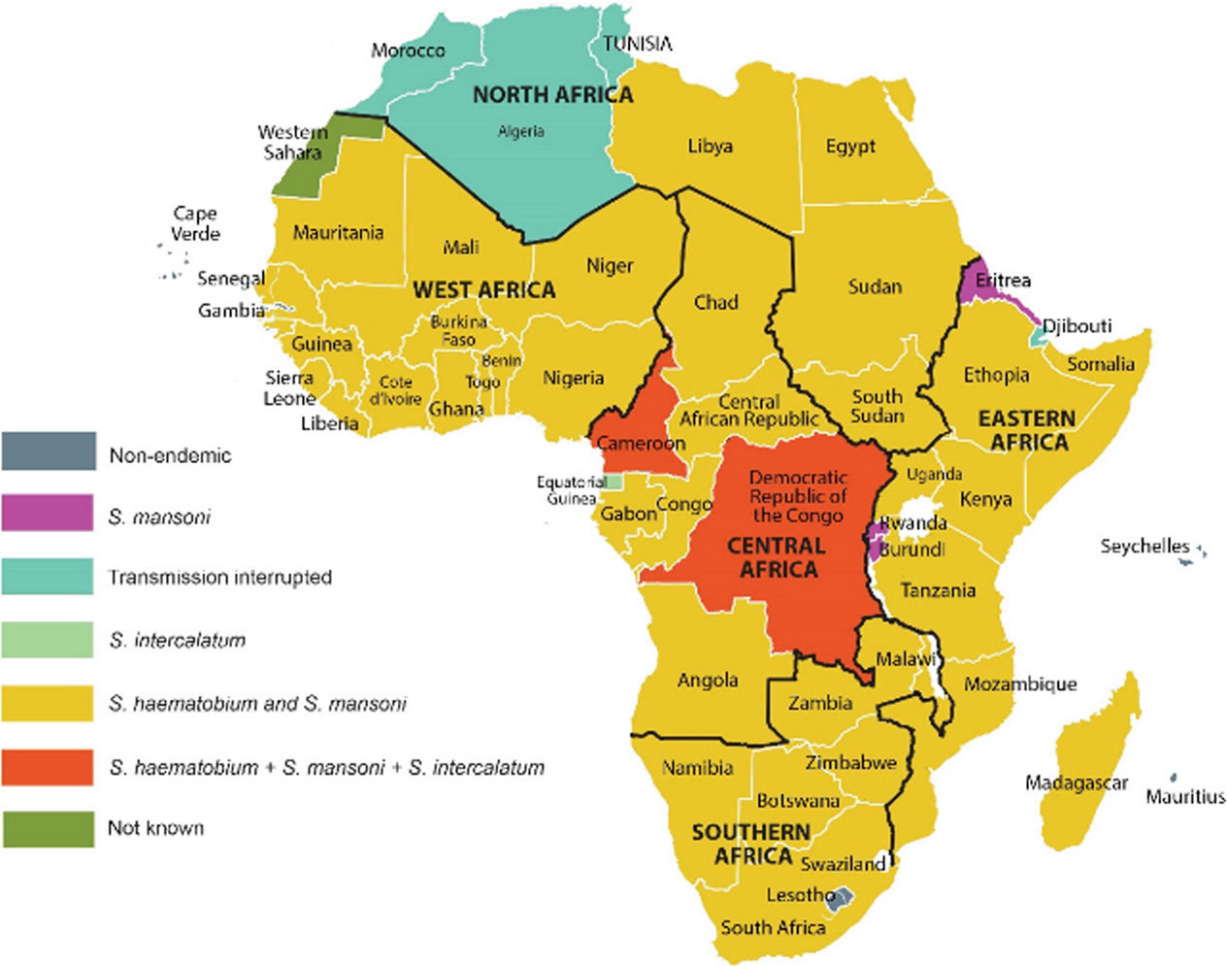
Schistosome infections in Africa. *Credit:* [47–49].

Owing to the severe public health burden of the disease, the control, prevention and elimination of schistosomiasis is a priority for the WHO, culminating in the implementation of varied intervention strategies to achieve corresponding set targets [4]. Paramount amongst those strategies is the MDA using PZQ targeting especially school-age children [41]. Through PC and other interventions such as effective freshwater snail control including mollusciciding, Tunisia and Morocco have interrupted transmission of schistosomiasis [42–44]. Egypt has made giant strides in schistosomiasis control and has recorded a consistent reduction in prevalence for years, whilst the disease elimination status of Algeria is still uncertain due to conflicting reports [45, 46]. Nonetheless, the incidence and prevalence of schistosomiasis continue to surge across Africa, especially south of the Sahara, as shown in Figure 1 [47–49]. Current prevalence data on schistosomiasis across Africa indicate that Nigeria and Tanzania rank first and second, respectively. At the same time, the Democratic Republic of Congo (DRC) and Ghana jointly share the third position [48, 50–52]. The increased incidence and prevalence of schistosomiasis in Africa may be attributed to inconsistent implementation of PC in endemic areas, lack of or inadequate WASH facilities and ineffective or lack of control of freshwater snails, amongst other factors.

The WHO in February 2022 recommended an integrated approach towards the control and elimination of human schistosomiasis. This approach involves the extension of PC to all persons at risk from 2 years of age in areas with a ≥ 10% prevalence, treatment of all infected individuals in health facilities, water snail control and environmental management, improvement of WASH and behaviour change [4]. In sub-Saharan Africa, PC in endemic areas or communities within the implementation units has resulted in a 60% reduction in the prevalence of schistosomiasis over the past 20 years.

The COVID-19 pandemic has adversely impacted schistosomiasis control and elimination efforts in endemic regions, particularly affecting MDA with PZQ for school-age children. The pandemic resulted in the disruption of mass treatment campaigns and public health education, thereby significantly eroding the gains made in schistosomiasis control and prevention over the years. A mathematical model indicates that these interruptions could delay elimination goals by up to 2 years in moderate and some high-prevalence areas [53]. In China and Brazil, there was a notable decline in activities related to schistosomiasis control and elimination, including population surveys, diagnosis and treatment of positive cases [54, 55]. The situation was no different in many African countries with a significant burden of the disease. Consequently, these disruptions are likely to contribute to a resurgence of schistosomiasis, particularly in high-transmission settings [56]. Whilst efforts geared towards mitigating the impact of COVID-19 in most African countries were implemented, less to no attention was paid to sustaining the gains made in schistosomiasis control and prevention over the years. Many African countries suspended various interventions especially PC in endemic communities. Uganda and Zimbabwe with high-risk populations did not implement PC in 2021, whilst South Africa and Equatorial Guinea are yet to start PC for schistosomiasis [4].

The resource demands of the HIV/AIDS pandemic in sub-Saharan Africa constrained fiscal space for other infectious diseases like schistosomiasis during and beyond the Schistosomiasis Control Initiative (SCI) programme era. International funding initiatives focused on HIV treatment and prevention, leading to reallocations within limited national health budgets and donor portfolios. Schistosomiasis, despite its high burden, is perceived as less impactful and prioritized [52, 57]. For example, in Uganda, the expansion of HIV programmes reduced funding for schistosomiasis, resulting in gaps in MDA coverage and surveillance [58, 59]. Consequently, scaling up essential, relatively low-cost schistosomiasis interventions was often deprioritized, sustaining transmission and burden throughout the pandemic era [57].

Additionally, the negative impact of tuberculosis (TB), HIV/AIDS and malaria on many African countries has significantly hindered efforts to control and eliminate schistosomiasis [60, 61]. The burden of these diseases on the continent has resulted in high morbidity and mortality rates, particularly amongst vulnerable populations [62–64]. For instance, South Africa experiences a high burden of HIV and TB, with approximately 20% of individuals living with HIV and ranking third globally for new TB infections [64]. The advent of COVID-19 adversely affected HIV services, leading to reduced access to antiretroviral therapy (ART), a decline in TB case detection [65, 66] and interruptions in the distribution of insecticide-treated bed nets for malaria [62]. Health education, promotion, diagnosis and preventive care in schistosomiasis-endemic areas were equally disrupted. These challenges primarily stemmed from resource diversion, temporary suspensions of research and limited access to healthcare, thereby eroding previous gains [64, 67].

The prevention and control of schistosomiasis, particularly in hard-to-reach rural and island communities, have been challenging due to limited access to healthcare facilities, poverty and misconceptions about the disease. However, new technologies that facilitate the dissemination of essential health information represent a significant advancement. These technologies can enhance health education, promote healthy behaviours and improve healthcare delivery [68]. Tools such as the use of mobile devices and, until recently, social media are significantly contributing to the gains made in public health [69]. Rural and isolated populations can now be reached more effectively, with health education messages delivered in local languages for better understanding. Whilst these technologies present valuable opportunities for health communicators, they also pose challenges in ensuring the effective communication of critical health information [68, 70].

### 1.7. Rationale of the Study

The rationale of this review is to assess the effectiveness of the various schistosomiasis control interventions implemented across Africa and make continent-specific recommendations necessary to meet the WHO's 2030 target.

## 2. Methods

### 2.1. Search Strategy and Selection Process

**Figure 2:**
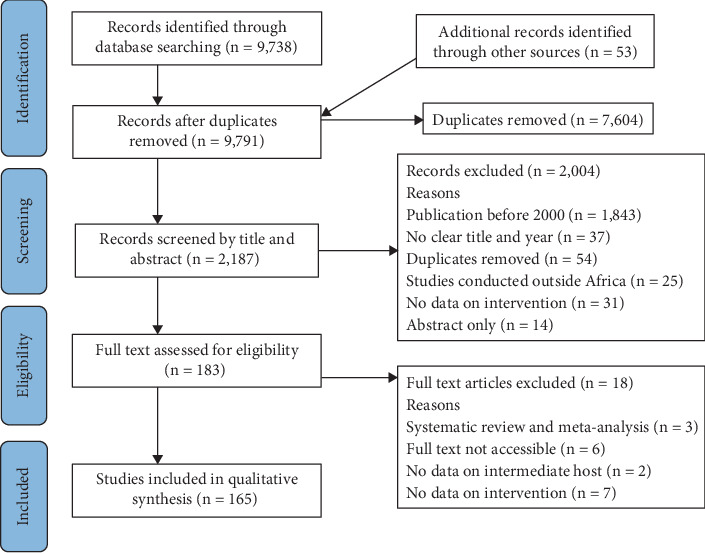
PRISMA flow chart showing the eligibility criteria for the studies.

A systematic literature search of studies was performed using the following MeSH (Medical Subject Heading) terms: ‘Human schistosomiasis control', ‘Animal schistosomiasis in Africa', ‘Schistosomiasis interventions', ‘MDA', ‘Schistosomiasis', ‘Bilharzia', ‘*Schistosoma*', ‘Freshwater snail control', ‘NTDs', ‘Elimination of NTDs', ‘Neglected tropical parasites' and ‘Schistosomiasis treatment' which was conducted according to the Preferred Reporting Items for Systematic Reviews and Meta-Analyses (PRISMA). Systematic computer-aided literature search was conducted using six English language databases, namely, African Journals Online, ScienceDirect, PubMed, WHO Database, Cochrane Library and Web of Science databases published from January 1, 2000, to August 30, 2023. Only published full articles available in the English Language were considered and analyzed. All relevant papers were thoroughly screened and reviewed according to the eligibility criteria (Figure 2). Duplicates were removed, and published articles on any intervention in relation to schistosomiasis control in Africa as a way of breaking the transmission were considered relevant and included in this study. The quality of the individual studies and the data underlying the publications was assessed prior to inclusion in this review.

### 2.2. Eligibility Criteria

Studies on human schistosomiasis, control and treatment methods at the local, regional or country levels were included. Studies on nonhuman *Schistosoma* infections, treatment outcomes outside Africa, conference abstracts, editorials, commentaries, case reports, protocols and narrative reviews were excluded in this review. No restriction whatsoever was placed on the age, sex, geographic location and education level of the study population, provided the study was conducted in Africa.

### 2.3. Identification of Studies and Data Extraction

The screening was conducted in a stepwise process. The initial screening of studies was done based on the title and abstract of retrieved articles by three coauthors (CYD, JPK and IA). The same three authors (CYD, JPK and IA) conducted full-text assessment of included studies when the abstracts were deemed insufficient to draw conclusions. Four independent senior coauthors (GBK, PBT, IA and PFA) constituted the reviewer panel, assessed the quality of individual studies and resolved by consensus any uncertainties or disagreements between the three full-text assessors on the inclusion of an article. The extraction of relevant data from each paper after full-text screening was summarized on data extraction forms. The full-text assessment evaluated and recorded lead author's name, country of origin, study design, study settings, sample size, participants characteristics/recruitment, schistosomiasis intervention reported, data analysis, key findings, conclusions and recommendations. For studies that were excluded, reasons for the exclusion were recorded. For this review, data on prevalence, distribution, treatment or control method, demographics and year of the study, as well as the study design, were vital. Studies on WASH intervention in relation to *Schistosoma* spp. infection control were included. Schistosomiasis control measures adopted by different countries in Africa were also critically examined for their effectiveness.

### 2.4. Data Synthesis

As a result of heterogeneity in the study design, study settings, study population and the nature of intervention, a thorough narrative synthesis was done to address the objective of the reviewers. Findings of the studies were tabulated, highlighting the prevalence, target population, population size, nature of intervention, study period and year the study was published, amongst others.

### 2.5. Patient and Public Involvement

Due to the nature of the study as a systematic review, no patients or the public were involved directly or indirectly in the conceptualization or conduct of this study.

## 3. Results

### 3.1. Search Results and Eligible Studies

A total of 9791 articles related to schistosomiasis in Africa were retrieved. Of these, 7604 were duplicates and therefore removed. After a thorough screening of the titles and abstracts, 183 articles remained for full-text assessment for eligibility. Following this, 165 articles were included in this systematic review. The PRISMA flow chart in Figure 2 shows the detailed screening and selection process.

The control and subsequent elimination of schistosomiasis in Africa needs a comprehensive multisectoral approach to interrupt transmission [71]. The integration of the One Health approach, an emerging public health methodology, is crucial for controlling neglected tropical diseases by targeting vulnerabilities across human, environmental, and animal interfaces that perpetuate such diseases in Africa, thereby leveraging synergistic benefits for schistosomiasis control [15]. Over the years, schistosomiasis control and prevention have been achieved by adopting pharmacological interventions such as MDA using PZQ and mollusciciding. The lack of adequate consistent flow of funding for the MDA against schistosomiasis is a major bottleneck in its control strategy. Most countries in Africa depend on development partners such as UNICEF, World Food Programme, USAID and Global Network for NTDs for financial support to undertake any such public health interventions. These donor-dependent supports are unsustainable, uncoordinated or narrow in scope, focusing primarily on MDA to school-age children. For the success of any schistosomiasis control measure, complementing the pharmacological intervention with nonpharmacological strategies is ideal for effective results. A control strategy backed by strong scientific study to understand the burden, distribution, reservoir hosts/animals, intermediate freshwater snail hosts, human behaviour, availability and appropriate use of WASH facilities will yield the desired outcome (Table 1).

### 3.2. Assessment of Interventions Across Africa

**Figure 3:**
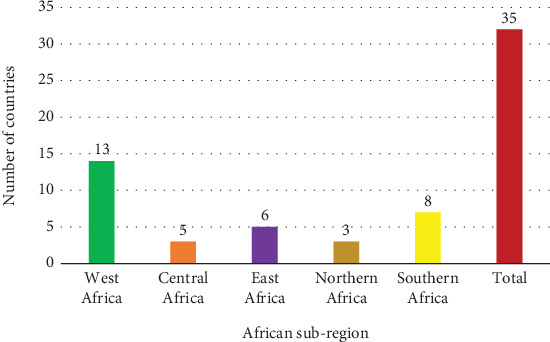
Subregional distribution of countries conducting schistosomiasis control interventions in this review.

High burden of schistosomiasis exists in a significant proportion of the 54 countries in Africa; 35 of them were captured in the 165 publications reviewed in this study, with varied (Table 1) and are distributed according to subregions, most of which are in West Africa and the least in Central and Northern Africa (Figure 3).

Although the WHO recommends a multifaceted approach to addressing the menace of urinary and intestinal schistosomiasis, the literature reviewed in this study indicates that a significant number of African countries rely predominantly on a single intervention—MDA by using PZQ—to control the disease. This approach is categorized as a pharmacological intervention, whilst any preventive or control strategy other than MDA is classified as nonpharmacological intervention.

**Figure 4:**
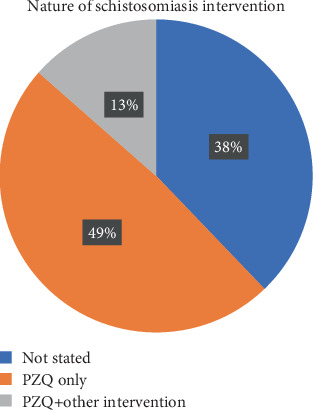
Proportion of countries implementing single or multiple schistosomiasis control intervention(s). MDA is mass drug administration (using praziquantel).

PZQ administration for preventive and curative purposes remains the single most implemented control measure across the 35 countries surveyed in this study. PZQ administration was either the only control strategy (18/35; 51.4%) or together with one or more other interventions (5/35; 14.3%). Studies conducted in 14 countries did not indicate the nature of schistosomiasis intervention (Figure 4).

The current study showed that studies on schistosomiasis in Africa or control interventions are funded and supported by the WHO and other international research initiatives (81/165; 49.1%), national governments (29/165; 17.6%) or private researchers (55/165; 33.3%).

## 4. Discussion

Despite significant efforts aimed at reducing the burden of schistosomiasis in Africa, the disease remains endemic on the continent, resulting in poor health outcomes amongst at-risk populations in resource-limited communities. We identified and categorized various schistosomiasis control measures implemented across Africa, including both pharmacological and nonpharmacological approaches. We assessed their effectiveness and provided continent-specific recommendations aimed at improving the chances of achieving the WHO's target for complete elimination by the year 2030.

### 4.1. Pharmacological Intervention

PZQ is recommended for the treatment and control of schistosomiasis caused by the five known *Schistosoma* species [48]. One-off oral PZQ (40 mg/kg of body weight) dose is commonly administered to at-risk populations in endemic areas. It is generally affordable and safe to use by both children and adults. Reinfection after PZQ administration is possible and, in areas of high endemicity, a single dose was found ineffective in complete parasite clearance. The MDA using PZQ programmes has often excluded preschoolchildren, asymptomatic carriers and adults [234]. These individuals become viable reservoirs for the transmission of the parasite. Some challenges to attaining the 100% coverage of the target populations during MDA include low enrolment of pupils in schools across Africa [87], infective larval stages of intermediate snail hosts, adult worms that persist after treatment [235] and immature worms in human hosts that escape treatment [236]. Currently, schistosomiasis cannot be prevented by vaccination as no vaccine has been developed or certified for human use by the WHO [237].

A study conducted by Nkengni et al. [238] revealed a significant decline in the prevalence of *S. haematobium* infections (34.2%) in Loum, Cameroon, compared to 62.8% in 2003, as was reported by Tchuem-Tchuente et al. [239]. The decline has been attributed to effective MDA using PZQ and increased awareness amongst schoolchildren and teachers/guardians about the risk factors of schistosomiasis. In Ghana, school-based PC using PZQ was implemented in 2008, 2010, 2011, 2012, 2014 and 2015, achieving coverage rates of 70%–85%. This control measure is based on WHO recommendations, focusing on reducing childhood morbidity through the annual administration of PZQ [141, 143]. Expanding the MDA with PZQ to include preschool-age children (PSAC) and adults could enhance schistosomiasis control, as the nonschool cohorts serve as reservoirs for disease transmission [240, 241]. That notwithstanding, results from the SCI highlight complexities associated with MDA using PZQ over extended periods. Although Ghana generally has low–moderate endemicity of schistosomiasis, a sharp increase in prevalence was reported between 2016 and 2019 in selected communities within the Brong Ahafo and Northern regions. This changed their endemicity from low to high [3, 242]. It is unclear what might have contributed to the high prevalence in these communities over the period.

Significant progress in controlling schistosomiasis in sub-Saharan Africa includes near completion of disease mapping by 2016 in 41 of 47 WHO African region countries, led by the WHO Regional Office for Africa (AFRO) and funded by the Bill and Melinda Gates Foundation [243]. With support from major donors and pharmaceutical partners, PC treatments rose from 7 million in 2006 to over 52 million by 2014, increasing coverage from 5.47% to 20.13% [244]. Consequently, treatment coverage for school-age children surged from 5.47% in 2006 to an estimated 41% by 2015, demonstrating substantial advancement towards control and elimination goals. Shifting from schistosomiasis control to elimination requires strategic changes. The current interventions, primarily for morbidity control or public health elimination [245], are insufficient for interrupting transmission. Achieving this goal necessitates intensified strategies focused on reducing transmission and preventing reinfection. Key challenges include implementing expanded interventions, improving WASH, securing sustained funding, strengthening surveillance and health systems and enhancing monitoring and evaluation [246].

The effectiveness of any pharmacological intervention such as MDA using PZQ would be seen when a comprehensive strategy has been adopted to cover PSAC, school-age children and adults. In this way, no segment of an endemic community will be left out and serve as a reservoir for transmission or reinfection. According to Liu et al. [236], PZQ remains effective for the treatment of both acute and chronic schistosomiasis, but its efficacy is enhanced when multiple doses are administered. They further opined that the use of artemether or artesunate with 1- or 2-week intervals as PC proves effective. Nonetheless, PZQ and artemether or artesunate in combination are more efficacious compared to PZQ monotherapy.

### 4.2. Nonpharmacological Interventions

#### 4.2.1. Intermediate Snail Host Control

Schistosomiasis is caused by several species of the trematode *Schistosoma*, namely, *S. haematobium*, *S. mansoni*, *S. intercalatum*, *S. japonicum*, *S. guineensis* and *S. mekongi* [1, 12]. Human schistosomiasis is caused by *S. haematobium*, *S. mansoni* and *S. japonicum.* The intermediate freshwater snail hosts responsible for human schistosomiasis, *Bulinus* sp., *Biomphalaria* sp. and *Oncomelania* sp., harbour the parasite. The *Bulinus* sp. is the intermediate host for *S. haematobium* [1], whilst *Biomphalaria* sp. is for *S. mansoni.* However, *Oncomelania* sp. serves as the intermediate host for *S. japonicum.*

The *Bulinus* freshwater snails are well distributed in Africa and the Middle East, with over 37 different species so far identified [247]. The distinct groups of the species named include *Bulinus africanus* group, *Bulinus reticulatus* group, *Bulinus forskalii* group and *Bulinus truncatus/tropicus* complex. Skin penetration by the cercariae occurs when humans have contact with infested freshwater. This leads to infection [248]. In Western Ethiopia, *Biomphalaria sudanica* and *Biomphalaria pfeifferi* are reported to have transmitted intestinal schistosomiasis, whilst *B. africanus* and *Bulinus abyssinicus* transmitted urinogenital schistosomiasis [249]. Collection of freshwater snails using a metal scoop net or handpicking is a way of controlling the abundance and distribution [239]. Morphological identification of *Bulinus* sp. is based on unique characteristics of the shell as described by Kristensen [250].

Most African countries have not prioritized freshwater snail control as a means of breaking schistosomiasis transmission. A case in point is Cameroon, where environmental management through snail control has not been done since 1975, although schistosomiasis is endemic in about 90 health districts [251, 252]. Seychelles, an archipelago in the Indian Ocean off East Africa, was the first to eliminate schistosomiasis in Africa [253]. The success story of Seychelles is a result of a consistent and comprehensive integrated approach encompassing education, PC, water snail control and WASH. Morocco is the only African country to have successfully interrupted the transmission of schistosomiasis since 2004 by integrating freshwater snail control into the elimination strategy. Niclosamide, a molluscicide very active against the intermediate freshwater snail hosts, is used by some countries in Africa, such as Kenya and Morocco [50, 254]. Tunisia and Algeria are awaiting WHO certification for successfully interrupting the transmission of schistosomiasis through an integrated control approach. In Zanzibar, the Elimination of Schistosomiasis Transmission project 2011–2017 targeted freshwater snail control through the application of niclosamide, and health promotion has been reported [254]. Although climate variation may have an impact on livelihoods, prolonged drought and rising temperatures have led to a significant reduction of the intermediate freshwater snail hosts due to the drying up of ponds and dams [229, 255, 256].

As part of the freshwater snail control strategy, the removal of vegetation suitable for the breeding of the intermediate snail hosts, environmental re-engineering by way of draining freshwater snail-infested ponds and dams, lining of canals with cement and restricting access to ponds and dams, amongst others, will lead to a drastic reduction in the incidence and prevalence of schistosomiasis [257, 258]. Until the control of the intermediate freshwater snail hosts is given priority attention, the roadmap to the elimination of schistosomiasis in Africa will be a remote possibility.

#### 4.2.2. Health Education/Training

Public health promotion through the education of people in both endemic and nonendemic areas and the training of healthcare staff on the signs and symptoms of schistosomiasis will lead to early case detection, diagnosis and treatment. Some studies conducted in Liberia, Nigeria, Zanzibar (Tanzania) and South Africa have reported low levels of knowledge amongst healthcare workers on FGS [258–260]. FGS is another grey area needing intensive public education on its endemicity, associated risk factors, clinical presentations and possible complications. Only a few studies have been conducted on FGS. Issues regarding the female genitalia are culturally and religiously sensitive in most communities in Africa. However, females, in the performance of household chores, access water bodies and end up being infected by the cercariae (larvae) of the parasite. The burden and distribution of FGS are largely unknown, posing a challenge to any robust elimination strategy. Blood in the urine of females (at puberty age) who are not menstruating is either thought to be menstrual blood, spotting or bleeding in-between period or a secondary sexual characteristic rather than an unhealthy condition. Urogenital schistosomiasis was thought to be a male child's and not a girl's problem. Consequently, such cases had been wrongly diagnosed by clinicians without any successful health outcome. Intensive public education on schistosomiasis as well as FGS will lead to behavioural change, accurate diagnosis, treatment and prevention in order to improve the health and wellness of society [258, 260].

Additionally, the overlapping of the symptoms of urogenital schistosomiasis with other health conditions poses a serious challenge to accurate diagnosis and treatment and increases the risk of complications. In women, parasite eggs in the reproductive tract cause chronic inflammation, fibrosis, tubal adhesions and granuloma formation in the ovaries, fallopian tubes and uterus, leading to infertility and ectopic pregnancies [261]. In men, the infection can induce infertility through hormonal imbalances, testicular tissue damage and obstruction of the genital ducts, resulting in azoospermia (the absence of sperm in semen) [262].

Another setback identified is the unavailability of PZQ in health facilities in Nigeria except during deworming exercises [69]. In South Africa, PZQ is beyond the affordability of ordinary citizens since other efficacious generic WHO-recommended PZQ is not approved by the South African government. This is a serious hindrance to reducing the burden of the disease in the Eastern Cape and KwaZulu-Natal areas [260].

Low coverage of PZQ administration in some endemic areas was caused by unfounded rumours that created mistrust and the fear of potential side effects (although transient), as reported in Kenya [263], Cote d'Ivoire [264] and Tanzania [265]. Furthermore, in Tanzania and Uganda, a great deal of children and adults refused treatment because of the drug's strong odour and enormous size [266, 267]. The involvement of key stakeholders (parents/guardians, children and adults) in mass PZQ administration will dispel any distrust, misinformation and myths.

#### 4.2.3. WASH

The quality of water and sanitation and the state of hygiene greatly impact the transmission of waterborne diseases including schistosomiasis. People who have access to safe water and practice good sanitation are less infected with schistosomiasis [268]. Schistosomiasis is endemic in resource-poor settings and hard-to-reach areas where WASH facilities are either nonexistent or inadequate [28, 268]. In such resource-poor settings, access to potable and safe water for recreational or agricultural activities is lacking; hence, humans become infected as a result of contact with a freshwater body infested with the intermediate snail hosts [1, 269].

Provision of WASH facilities is one of the SDGs (Goal 6) of the United Nations [270]. The provision of adequate WASH facilities will not only help reduce the NTD burden in endemic communities but also improve child and maternal health and control or prevent gastroenteric illnesses such as diarrhoea and vomiting [268–271]. Evidence suggests that a significant decline in schistosomiasis and other NTD cases in endemic communities was attributable to the provision of WASH facilities [267]. Children (especially below 5 years old) in resource-poor settings are the most vulnerable and stand the risk of getting diarrhoeal diseases if WASH facilities are lacking or not in adequate supply. Despite its importance, WASH interventions are not integrated in the control of schistosomiasis [254, 272].

Whilst improved access to clean water and sanitation is a critical intervention emphasized by WASH, a persistent lack of adequate funding and resources poses a significant challenge. Furthermore, the financial resources needed to create and maintain such WASH infrastructure are out of the financial possibility of any sub-Saharan African country where schistosomiasis is endemic. This is why, as it results from their study, the schistosomiasis control programme worldwide concentrates on drug administration that is achievable with the limited resources available and not on WASH. Schistosomiasis is closely associated with poverty; therefore, the provision of potable water, adequate sanitation and hygiene facilities should form key components of any control strategy. Although some regions in Africa have observed a reduction in the prevalence of schistosomiasis, transmission continues, threatening to undermine the progress achieved. Expanding an integrated control strategy is essential for achieving total elimination [273].

#### 4.2.4. Animal Schistosomiasis

The One Health concept provides a comprehensive approach to disrupting the transmission of animal schistosomiasis by recognizing the interconnectedness of humans, animals and the environment. In areas where animal schistosomiasis is prevalent, a control strategy should focus on livestock (such as rodents, cattle, sheep, pigs and goats) that serve as reservoirs for schistosomes. This integrated approach not only emphasizes the treatment of infections in humans but also addresses animal health and promotes environmental sanitation. This strategy has the potential to mitigate the risk of transmission to other animals and potentially to humans.

A study conducted in Ethiopia by Alehegne and Mitiku [274] revealed a significant proportion of rodents serving as reservoirs of *S. mansoni.* Animals or rodents serving as reservoirs to sustain the transmission of schistosomiasis are an emerging phenomenon that needs to be scientifically explored. It has been established that *S. mansoni* infection increased in rodents due to their closeness to human habitations and possible direct effect on human infections. These rodents are a potential source of transmission of the parasite to humans or other animals. Separate studies conducted in the Upper East Region and Coastal Savannah of Ghana revealed that livestock (cattle and pigs) harbour *S. mansoni* [13, 14]. The spillover effect of new *Schistosoma* hybrids from animal hosts may have serious implications for the control and successful elimination. The hybrid genes may be more virulent, thereby posing a serious health risk to humans. In Uganda and Zimbabwe, prolonged drought has diminished freshwater snail populations and consequently schistosome infections. This may be positive but has a devastating effect on livelihoods and the general health of the people. Although animal schistosomiasis is gradually becoming a public health concern, in this review, we did not come across any intervention that has been undertaken to control or eliminate it.

Overall, whilst the use of MDA with PZQ has significantly reduced the burden of schistosomiasis in Africa, particularly in endemic and hyperendemic areas, more work is needed to achieve total elimination. This requires consistent implementation of nonpharmacological control measures—such as controlling intermediate snail hosts, providing health education and training, improving WASH and addressing animal schistosomiasis. This integrated schistosomiasis control strategy, supported by national governments, has yielded positive results in Seychelles, Morocco, Tunisia and the People's Republic of China. The strategy combines environmental approaches—such as improved sanitation, agricultural and hydrological development and management—with chemical-based drug treatments and mollusciciding. Mollusciciding is a vital component of the integrated strategy for the control and elimination of schistosomiasis, complementing PC and other interventions. Niclosamide (Bayluscide) has proven effective in reducing freshwater snail populations, disease parameters and water infectivity. The use of low-dose applications may improve community acceptance by minimizing impacts on nontarget aquatic organisms whilst preserving water quality [275]. When implemented effectively, this approach targets various phases of the parasite transmission cycle [276]. However, effective community engagement is vital for developing and implementing a successful schistosomiasis control strategy using molluscicides. Whilst the use of chemical-based molluscicides can significantly reduce prevalence and incidence, it does not eliminate transmission [277]. It has also been opined that incorporating snail control into MDA is a cost-effective strategy for managing schistosomiasis, particularly in areas with high prevalence [278]. However, many African countries either do not implement complementary control strategies or lack effective implementation of these measures. The continued reliance on MDA using PZQ as the primary strategy for controlling schistosomiasis hinders progress in shifting the focus from control to elimination by integrating both pharmacological and nonpharmacological interventions.

#### 4.2.5. Strengths and Weaknesses of This Review

This review is the result of a comprehensive search using general and inclusive terms with a well-stratified synthesis of available data. Relevant data on the control, prevention and elimination of schistosomiasis in both humans and animals were included. The limitation of this study is the exclusion of studies published in languages other than English, leading to accidental omission of valuable untranslated research articles in this review. Studies conducted outside Africa which may be valuable to this review were omitted.

## 5. Conclusion and Recommendation

Inadequate coordination of schistosomiasis control programmes at the national, regional or continental levels is a major setback to achieving elimination status. Across Africa, mass administration of a one-off dose of PZQ, especially to school-age children in a school-based system, is the main control strategy. However, not every school-age child is enrolled in school due to a lack of access or economic reasons. PSAC and adults in endemic areas are often not targeted during MDA, although some may serve as reservoirs of the parasite. It is worth noting that MDA programmes in most African countries are donor-driven, leading to inconsistency and narrowness in scope. These ad hoc measures are less effective and should be replaced with a well-thought-out integrated approach, such as nonpharmacological strategies, to reduce the burden or eliminate schistosomiasis in Africa. No coordinated integrated schistosomiasis control strategy has so far been adopted across Africa. In this review, 20/32 (62.5%) countries in Africa implemented a single intervention, namely, MDA with PZQ, to achieve the elimination target by 2030. The integration of pharmacological (drug administration) and nonpharmacological (freshwater snail control, human behaviour change, health education, provision of safe water and hygiene facilities) measures will significantly improve health outcomes and the overall wellness of at-risk populations. Additionally, schistosomiasis-related studies integrating WASH interventions will provide insight into the extent of shortfalls and the comprehensiveness of the required interventions. We recommend that African governments include disease control in national budgets to support needs assessments and select model sites for integrated interventions in collaboration with research institutions to document best practices. When development partners or donor support is available, the programme can be scaled up and adapted for other endemic communities.

## Figures and Tables

**Table 1:** Schistosomiasis and nature of intervention across Africa.

**Country**	**% prevalence**		**Target population/years**	**Targeted population size**	**Nature of intervention**	**Study period**	**Year study published**	**Reference**
	***S. haematobium***	***S. mansoni***						
Angola	12.6	0.9	SAC (10–14)	31,938	MDA (praziquantel), health education	October 2018 and July–December 2019	2022	[72]
	10.0–21.7	Not stated	TP (0–80)	3339	MDA (praziquantel)	May and August 2010	2012	[73]
	71.7 (215/300)	Not stated	TP (15–75)	300	Not stated	November 2007–February 2008	2015	[74]
	70.1 (47/67; baseline); 53.7 (36/67; first follow-up); 76.1 (51/67; second follow-up)	Not stated	SAC (2–15)	198	Praziquantel	December 2012–December 2013	2019	[75]
	13.6	21.2	SAC (9–13)	17,093	MDA (praziquantel)	March 23–August 12, 2014	2022	[76]
	61.2 (785/1283)	Not stated	SAC (5-11)	1283	MDA (praziquantel)	March 2013 and February 2014	2015	[77]

Benin	0.8–56.0	0.4–46	SAC (8–14)	7–500	MDA (praziquantel)	2014	2016	[78]
	17.60	2.45	SAC (8–14)	19,250	MDA (praziquantel)	2013–2015	2019	[79]
	22.9–29.4	Not stated	SAC (7–16)	1585	Praziquantel	May–September 2010 and September 2010–June 2012	2014	[80]
	2.7 and 7.6	Not stated	SAC (9–14)	92	Praziquantel	December 2010	2012	[81]
	34.5 (48/139), 13.5 (18/133) and 11.9 (17/143)	Not stated	SAC (8–14)	415	Praziquantel	May–July 2022	2023	[82]
	30.1	Not stated	SAC (≥ 18)	734	Praziquantel	July 9–31, 2023	2023	[83]

Botswana	8.7	0.6	SAC (6–13)	1,611	MDA (praziquantel)	2022	2023	[84]

Burkina Faso	0.0 (0/160)–56.3 (90/160)	8.8 (42/480)	SAC (7–11)	3514	MDA (praziquantel)	2013	2016	[85]
	0.0	42.1 (96/228)	SAC (1–5)	228	Praziquantel	February–March 2020	2021	[86]
	59.6–7.7	Not stated	SAC (6–14)	1727	MDA (praziquantel)	2004–2005, 2005–2006	2008	[87]
	11.8	Not stated	SAC (7–11)	3324	MDA (praziquantel)	November 2007 and February 2008	2011	[88]

Cameroon	50.6	Not stated	TP^a^	338	MDA (praziquantel)	June 2016	2017	[89]
	0.3	19.8	TP (3–78)	369	Not stated	September 2014 and May 2015	2019	[90]
	31.5	Not stated	5–75	1029	Praziquantel	June and August 2018	2020	[91]
	32.6	Not stated	SAC (5–15)	389	Praziquantel	April–May 2018	2021	[92]
	58.6	Not stated	5–89	304	Praziquantel	2020	2021	[93]
	13.0	Not stated	SAC (4–14)	638	Praziquantel	March and June 2015	2021	[94]

Chad	55.0 (6646/11,832)	Not stated	SAC (5–15)	11,832	MDA (praziquantel)	2015–2019	2022	[95]
	50.5	Not stated	1–15	400	Praziquantel	July 15 and August 30, 2017	2021	[96]
	24.9 (467/1875)	Not stated	1–14	1875	Praziquantel	March 2015 and March 2016	2019	[97]
	39.2 (35.3–54.9)	8.6	SAC (≤ 18)	258	Praziquantel	January 2019	2020	[98]

Cote d'Ivoire	0.3 (3/1248)	3.5 (42/1202)	SAC (5–15)	728	MDA (praziquantel)	March 2015	2018	[99]
	2.6	9.5	SAC(9–12)	274	MDA (praziquantel)	October and December 2016	2020	[100]
	10.0	29.1	SAC (4–15)	353	Praziquantel	April–September 2001	2017	[101]
	14.0 (166/1187)	Not stated	SAC (5–14)	1187	Not stated	January–April 2018	2019	[9]
	7.0; 2.2; 3.4	2.2; 0.8; 3.8	≤ 5 ≥ 25	750	Not stated	August–September 2014	2019	[102]
	Not stated	31.4 and 62.9	PSAC^c^ (1–6)	350	Praziquantel	June 2016–December 2017; July–September 2018	2021	[103]
	10.6; 16.2; 9.4	Not stated	5–8; 9–12; 20–55	12,348	Praziquantel	9–28 November 2015	2021	[104]
	5.4 and 2.7	0.3	6–96	742	Praziquantel	August 2018	2023	[105]

Democratic Republic of Congo	41.0	36.3	SAC (5–15)	480	MDA (praziquantel)	June–August 2021	2023	[106]
	Not stated	59.2 and 65.7	TP (6– ≥ 50)	949	MDA (praziquantel)	June and September 2017	2020	[107]
	70.0	58.0	1–80	314	Not stated	2009	2022	[108]
	17.4	Not stated	Pregnant women (18– ≥ 35)	367	Praziquantel	October 2016 and March 2017	2019	[109]
	36.3	38.4	SAC (5–15)	480	MDA	June–August 2021	2023	[106]
	Not stated	73.1	TP (1–50)	1044	Praziquantel	2017	2020	[110]
	Not present	8.9	SAC (7–13)	526	MDA (praziquantel)	April–May 2016	2017	[111]

Egypt	1.5	1.8	Patients	400	Praziquantel	November 2017 and August 2018	2019	[112]
	37.3 (50/134)	Not stated	Patients from urology outpatient clinic	134	MDA (praziquantel)	October 2018–February 2019	2022	[113]
	24.0 (568/2371)	Not stated	Adults (≤ 18, ≥ 18)	2371	Not stated	January 2016–December 2018	2020	[114]
	7.9	Not stated	1–70	1000	Not stated	2016	2017	[115]
	4.7	1.3	6 years to 40	1200	Not stated	January 2020–January 2021	2021	[116]
	Not stated	19.1	6–15	861	Not stated	July and November 2019	2022	[117]
	Not stated	1.5	SAC (10–12)	1100	Not stated	2018–2019	2020	[118]
	Not stated	12.4	Patients (22–68)	193	Not stated	August 2018 and January 2020	2021	[119]

Eswatini (Swaziland)	16.0 (32/200)	Not stated	SAC (10–15)	200	Not stated	April 16–May 15, 2015	2023	[120]

Ethiopia	Not stated	25.6 (204/798)	SAC (6–15)	798	MDA (praziquantel)	December 2017 and February 2018	2021	[121]
	Not stated	25.8 (815/3162)	SAC (5–15)	3162	MDA (praziquantel)	2018–2019	2020	[122]
	Not stated	9.3 (75/389)	SAC (5–15)	389	MDA (praziquantel)	April–May 2019	2020	[123]
	Not stated	28.7	SAC (7–17)	328	Praziquantel	March–April 2017	2020	[124]
	Not stated	15.2	SAC (7–15)	362	Praziquantel	November 2018–March 2019	2020	[125]
	Not stated	4.8	SAC (6–15)	421	Praziquantel	May–June 2021	2022	[126]
	Not stated	73.8	SAC (6–15)	492	Praziquantel	October 2018–September 2019	2022	[127]
	Not stated	52.1	SAC (5–18)	499	Praziquantel	2020–2021	2022	[128]
	0.3	3.5	5–15	153,238	MDA (praziquantel)	2013 and 2015	2020	[129]
	Not stated	33.5	SAC (7–16)	786	Praziquantel	January 21–February 21, 2018	2020	[130]
	Not stated	11.4 (34/298	SAC (5–19)	298	Praziquantel	January and March 2018	2020	[131]
	Not stated	53.9 (201/373)	SAC (5–19)	373	Praziquantel	2017–2020	2021	[132]

Gabon	43.1 (112/260)	Not stated	Volunteers	351	Noted stated	June 2016–February 2018	2019	[133]
	26.3	Not stated	SAC (5–19)	612	MDA (praziquantel)	April–July 2016	2021	[134]

Gambia	10.2	0.3	SAC (7–14)	2018	MDA (praziquantel)	2015	2021	[135]
	0.0–9.4	0.0–0.4	SAC (7–14)	10,434	MDA (praziquantel)	May 2015	2021	[136]
	28.7	1.5	TP (6–75)	195	Praziquantel	March–April 2017	2020	[137]

Ghana	25.3	Not stated	TP	140	MDA (praziquantel)	August 2021	2023	[138]
	24.8	Not stated	Women (20–49)	420	Not stated	Not stated	2011	[139]
	18.3 (77/420)	Not stated	SAC	420	Praziquantel	September 2016–March 2017	2020	[140]
	0.0–45.0	Not stated	SAC	900	MDA (praziquantel)	November 2–15, 2015	2019	[141]
	66.8 (135/202)	90.1 (163/181)	SAC (5–26)	202	MDA (praziquantel)	June 2012–September 2013	2019	[142]
	3.3–19.0	30.0–78.3	TP (0–75+)	658	MDA (praziquantel)	Not stated	2020	[143]
	76.8	Not stated	SAC (4–20)	112	MDA (praziquantel)	September–November 2018	2022	[144]
	12.8 (43/336)	Not stated	SAC (5–16)	336	MDA (praziquantel)	September–October 2018	2022	[145]
	10.4	Not stated	SAC (age not stated)	309	Not stated	March–July 2020	2021	[146]
	27.5 and 17.0	Not stated	SAC (6–20)	250	Not stated	January–May 2013	2016	[147]

Kenya	Not stated	17.4	SAC (9–10)	492	Community-directed intervention MDA (praziquantel)	2001	2012	[148]
	15.3 (69/451)	Not stated	≤ 10 ≥ 36	451	Praziquantel	February–March 2018	2020	[149]
	9.3	13	SAC (7–18)	3487	MDA (praziquantel)	May–June 2013	2014	[150]
	14.8 (baseline), 6.8 (midterm), 2.4 (endline)	2.1 (baseline), 1.5 (midterm), 1.7 (endline)	SAC	21,528 (baseline), 21,111 (midterm), 21,045 (endline)	MDA (praziquantel)	2012–2017	2019	[151]
	6.1	0.5	SAC (8–14)	27,850	Praziquantel	October–November 2020	2023	[152]

Liberia	71.0 (5/7)	Not stated	10–15	20,000	MDA (praziquantel)	January 2013–December 2017	2021	[153]
	50.0	Not stated	TP	Not stated	MDA (praziquantel)	2012	2012	[154]
	44.3 (258/582)	Not stated	Women (18 ≥)	582	Praziquantel	April–August 2021	2023	[155]

Madagascar	30.5	5.0	SAC (7–10)	1958	Not stated	October 2015	2016	[156]

Malawi	17.1	3.8	Adults (18–70)	376	MDA (praziquantel)	October 2017–December 2018	2021	[157]
	1.4–15.3	31.5 (1.7–60.0)	SAC (6–15)	520	MDA (praziquantel)	May–June 2019	2020	[158]

Mali	78.4	Not stated	TP (≤ 15 and ≥ 15)	8022	Community-directed intervention MDA (praziquantel)	October 2007–December 2008	2023	[159]
	0.0–20.8	Not stated	SAC (7–14)	1836	MDA	December 2014–2015 and April 2018	2021	[160]
	38.4 (844/2196)	5.6 (124/2196)	SAC (7–14)	2196	MDA (praziquantel)	March–April of 2004	2010	[161]

Mauritania	4.0 (86/2162)	Not stated	SAC (5–15)	2162	Not stated	September 2014 and May 2015	2017	[162]
	15.6 (48/307)		7–17	307	Not stated	2018	2019	[163]

Morocco	Eliminated	Eliminated	—	—	MDA (praziquantel), water snail control, health education, intersectoral collaboration	1960–2018	2020	[164]
	African immigrants from Mauritania (37%), Mali (18%) and Senegal (15%)^b^	Not stated	Adults	27 cases	A sustainable surveillance and control system to check imported cases	2005–2017	2020	[165]

Mozambique	60.5–38.8	Not stated	TP (9–12, 20–55)	81,167	MDA (praziquantel)	July and October 2011–2015	2017	[166]
	2.8	1.4	20–27	362	Praziquantel	2022	2023	[167]
	47.0	Not stated	SAC (7–22)	83,331	MDA (praziquantel)	August 2005–June 2007	2009	[168]

Namibia	9.2	39.2	SAC (3–19)	17,896	MDA (praziquantel)	November 2012 and November 2013	2015	[169]

Niger	9.5 (2134/22,364); 9.9 (2190/22,132); 4.9 (493/9955)	Not stated	SAC (5–8); SAC (9–12); adults (20–55)	167,500	MDA (praziquantel)	2011–2015	2020	[170]

Nigeria	4.1	Not stated	1–18	120	Not stated	2022	2023	[171]
	13.3	17.3	SAC (6–21)	340	Praziquantel	June 2020 to February 2021	2022	[172]
	23.2	Not stated	5–55	1404	Not stated	2017	2019	[173]
	28.9	9.5	5 ≥ 21	432	Praziquantel	September 2020	2022	[174]
	57.0	Not stated	1–64	384	Praziquantel	October and November 2019	2022	[175]
	19.0 (89/466)	9.0 (41/465)	4–19	466	MDA (praziquantel)	March 2018 and May 2019	2021	[176]
	Not stated	4.2 (35/829)	TP (1–80)	829	Praziquantel	May and June 2018	2021	[177]
	9.5 (10,349/108,472)	Not stated	SAC (5–16)	108,472	Not stated	November 2013 and May 2015	2019	[178]
	34.7 (104/300)	Not stated	SAC (6– ≥ 10)	300	Not stated	2016	2017	[179]
	55.0 (165/300)	Not stated	1–15	300	Not stated	September 2012	2014	[180]
	70.0 (80/120)	Not stated	TP	120	Not stated	October 2014–February 2015	2016	[181]
	21.3 (64/300)	Not stated	SAC (5–16)	300	MDA	May and September 2019	2020	[182]
	16.2 (57/353)	Not stated	SAC (4–16)	353	MDA (praziquantel)	June 2019–December 2019	2023	[183]
	11.5	Not stated	≤ 10 and ≥ 20	355	Not stated	November–December 2020	2022	[184]
	10.4	Not stated	10–20	2023	Not stated	October–November 2018	2021	[185]

Rwanda	Not stated	6.5 (265)	SAC (5–15)	4998	MDA (praziquantel)	April 2019	2023	[186]
	Not stated	36.1	3–17	19,371	MDA (praziquantel)	June 2014 to mid-July 2014	2020	[187]
	Not stated	24.0	PSAC^c^ (7–68 months)	4675	Praziquantel	2020–2021	2022	[188]
	9.5	Not present	SAC (1–4)	278	Praziquantel	August 2016	2019	[189]

Senegal	57.6 (121)	Not stated	7–15	210	MDA (praziquantel)	February–June 2009	2014	[190]
	77.1 (2016 baseline); 66.3 (2017 reinfection); 68.1 (2018 reinfection)	34.9 (2016 baseline); 13.8 (2017 reinfection); 25 (2018 reinfection)	SAC	1400	MDA (praziquantel)	2016 and 2018	2021	[191]

Sierra Leon	2.2	20.4	9–14	3632	MDA (praziquantel)	2016	2019	[192]
	Not stated	1.2	1– ≥ 18	815	Praziquantel	November 2019 and February 2020.	2022	[193]
	Not stated	16.3	SAC (10–12), adults	3685	MDA (praziquantel)	2012	2014	[194]
	Not stated	1.4 and 13.8	(PSAC^c^: 1–4; SAC 5–14 and 15–49)	3685	MDA (praziquantel)	May 2018	2022	[195]

South Africa	16.8 (353/2105)	Not stated	SAC (9–14)	2105	Praziquantel	March–December 2007	2018	[196]
	99.8 (135 372/135 627)	0.2 (255/135 627)	0– ≥ 40	135,627	MDA (praziquantel)	1 January 2011 and 31 December 2018	2021	[197]
	32.2	Not stated	SAC (10–12)	1241	MDA	January and November 2010	2020	[198]
	1.0	0.9	1–5	1143	MDA (praziquantel)	June–September 2018	2019	[199]
	36.9	Not stated	SAC (10–12)	726	Praziquantel	September 2009–November 2010	2016	[200]
	37.5 (120/320)	Not stated	SAC (10–15)	320	Praziquantel	June 2015 and March 2016	2017	[201]
	32 (312/970	Not stated	SAC (10–12)	1057	Praziquantel	September 2009–November 2010	2013	[202]

Sudan	5.2	0.1	SAC (7–15)	105,167	MDA (praziquantel)	December 2016–March 2017	2019	[203]
	35.6	2.6	TP (0– ≥ 30)	1138	MDA (praziquantel)	January–February 2014	2019	[204]
	28.5	0.4	TP (≤ 15 and ≥ 15)	78,615	Integrated control programme (MDA praziquantel, health education, WASH facility)	2009–2011	2015	[205]
	45.0	5.9	5–15	338	Praziquantel	April 2009–February 2010	2014	[206]
	12.9	3.0	6–17	170	Not stated	November 2017–February 2018	2018	[207]

Tanzania	6.1	8.7	SAC (9–13)	8,698	MDA (praziquantel)	March and May 2021.	2022	[51]
	Not stated	90.6	7–19	830	MDA (praziquantel)	February and May 2017	2020	[208]
	32.5	Not stated	SAC (8–16)	884	MDA (praziquantel)	2023	2023	[209]
	Not stated	11 (36/328)	Adults (18–55)	328	Praziquantel	July and August 2020	2022	[210]
	5.4 (children); 2.7 (adults)	Not stated	9–12; 20–55	39,207; 18,473	Praziquantel	February–June 2013–2016	2018	[211]
	16.8		6–14	250	Praziquantel	October 2015 to July 2017	2018	[212]
	29	Not stated	6–19	879	Praziquantel	2006–2009	2011	[213]
	3.9–0.4	Not stated	TP (SAC ≤ 8 ≥ 13; adults ≤ 19 ≥ 56)	19,293	MDA (praziquantel)	2011–2020	2021	[214]

Togo	4.2	0.8	SAC (5–14)	7248	MDA (praziquantel)	8 November and 4 December 2021	2023	[215]
	Not stated	5.0	SAC (6–9)	17,100	MDA (praziquantel)	February 15 to March 31, 2015	2018	[216]

Uganda	Not stated	25.6	2–50+	9183	MDA (praziquantel)	October–December 2016–2017	2019	[217]
	Not stated	88.6	7–76	446	Praziquantel	2012	2013	[218]
	Not stated	96	6–14	55	Praziquantel	February 2019	2020	[219]
	Not stated	71.0	TP (PSAC: 9 months–4.9; SAC: 5–14.9; adults: ≥ 15)	381	Praziquantel	March and November 2017	2021	[220]

Zambia	9.7 (41/421)	Not stated	SAC (≤ 10–≥ 16)	421	Not stated	2019–30 January 2020	2022	[221]
	3.5	Not stated	10–16	173	Not stated	2020–2021	2022	[222]
	28.6	Not stated	9–16	975	Praziquantel	2007–2015	2018	[223]
	61 (90/147)	Not stated	7–14	147	Praziquantel	2017	2020	[224]
	83.3	Not stated	6–15	562	MDA (praziquantel)	October 2008–November 2009	2012	[225]
	11.4 (62/542)	Not stated	6–17	542	Not stated	2019	2021	[226]
	13.3	Not stated	5–17	354	(Praziquantel)	November 2020 and February 2021	2023	[227]
	Not stated	42.4 (304/719)	TP (7–50)	754	MDA (praziquantel)	2013	2014	[228]

Zimbabwe	18.0	7.2	SAC (10–15)	13,195	Not stated	September 2010 and August 2011	2014	[229]
	15.4 (132/860)	Not stated	PSAC and caregivers (≤ 5–≥ 15	860	Praziquantel	February 2016	2019	[230]
	31.7–0.0	4.6–0.0	SAC (6–15)	7529	MDA (praziquantel)	September 2012–November 2017	2020	[231]
	35 (145/415)	Not stated	PSAC^c^ (1–5)	465	Praziquantel	2018	2020	[232]
	23.1–0.5	9.0 (21/233)	SAC (7–13)	212	MDA (praziquantel)	2017	2019	[233]

Abbreviations: SAC, school-age children; TP, total population.

^a^TP: PSAC ≤ 6 years, SAC ≥ 6 years, adults > 16 years.

^b^Imported cases (PSAC, SAC, adults and unknown).

^c^PSAC (preschool-age children or children under 5 years are at the moment excluded from praziquantel treatment).

## Data Availability

Data sharing is not applicable to this article. No new data was generated or analyzed in this study.

## Nomenclature

MDA: mass drug administration
NTDs: neglected tropical diseases
PC: preventive chemotherapy
PZQ: praziquantel
SAC: school-age children
SDGs: Sustainable Development Goals
WASH: water, sanitation and hygiene
WHO: World Health Organization
